# Supplementary material for: Effectiveness, Timing and Procedural Aspects of Cognitive Behavioral Therapy after Deep Brain Stimulation for Therapy-Resistant Obsessive Compulsive Disorder: A Systematic Review
Source: J Clin Med. 2020 Jul 26;9(8):2383. doi: 10.3390/jcm9082383 (PMC7464329; doi:10.3390/jcm9082383)
Supplement: Supplementary file 1 [file jcm-09-02383-s001.pdf]

Table S1. Quality ratings of the included studies according to the Evidence Project risk of bias tool (Kennedy 2019).

study	cohort	Control or comparison group	Pre-/post-intervention data	Random assignment of participants to the intervention	Random selection of participants for assessment	Minimum follow-up rate 80%	Comparison groups equivalent on sociodemographics	Comparison groups equivalent at baseline on disclosure
Denys et al 2010*	Y	N	Y	N	N	Y	n.a.	n.a.
Mantione et al 2014*	Y	N	Y	N	N	Y	n.a.	n.a.
Tyagi et al 2019	Y	N	Y	N	N	Y	n.a.	n.a.
Greenberg et al 2006	Y	N	N	N	N	Y	n.a.	n.a.
Abelson et al 2005	N	N	N	N	N	Y	n.a.	n.a.

Studies were rated for their methodology with respect to CBT and not with respect to the DBS. * These two studies are analyses from the same clinical trial. Y = yes, N = no, n.a = not applicable.
